# Mapping the evidence on post-intensive care syndrome in paediatric populations: A scoping review protocol

**DOI:** 10.1371/journal.pone.0338293

**Published:** 2026-03-25

**Authors:** Shoheb Hassan, Ali Hassan

**Affiliations:** 1 Aston Medical School, College of Health and Life Sciences, Aston University, Birmingham, United Kingdom; 2 University of Exeter Medical School, Faculty of Health and Life Sciences, University of Exeter, Exeter, United Kingdom; University of Porto Faculty of Medicine: Universidade do Porto Faculdade de Medicina, PORTUGAL

## Abstract

**Objective:**

This scoping review aims to comprehensively map the primary literature on post-intensive care syndrome in paediatric populations (PICS-p), across all recognised PICS-p domains, with a focus on diagnostic methodologies, preventive and therapeutic interventions, and reported post-ICU outcomes among children aged 1 month–18 years.

**Introduction:**

Post-intensive care syndrome (PICS) encompasses new or worsening physical, cognitive, psychological, or social impairments emerging after critical illness. While extensively studied in adults, its paediatric counterpart, PICS-p, remains under-recognised and inconsistently characterised. Children surviving paediatric intensive care may face long-term functional limitations, developmental challenges, and psychosocial difficulties, with substantial implications for families and caregivers. Existing evidence is fragmented across diverse clinical contexts, age groups, and outcome measures, and no comprehensive synthesis has mapped how PICS-p is assessed, managed, or monitored. A structured overview is needed to clarify current practice, highlight gaps, and inform future research and clinical pathways.

**Inclusion criteria:**

We will include primary clinical studies involving paediatric patients aged 1 month–18 years who have survived admission to a paediatric or specialised intensive care unit. Eligible studies may evaluate diagnostic or screening tools, preventive or therapeutic interventions, or longitudinal outcomes related to PICS-p. Studies including mixed-age populations will be incorporated only when paediatric data are reported separately or can be disaggregated. Grey literature reporting primary clinical data will be included. Exclusions apply to neonatal-only cohorts, adult-only studies, abstract-only publications, non-clinical reports, and studies focusing solely on in-ICU outcomes without post-discharge assessment. Only English-language studies published from 1 January 2000 onward will be considered.

**Methods:**

Following PRISMA-ScR and Joanna Briggs Institute guidance, this scoping review will conduct systematic searches across PubMed, Scopus, the Cochrane Library, ProQuest, CINAHL, medRxiv, and major clinical trial registries. Searches will be limited to English-language studies published from 1 January 2000 to the date of search. All records will be deduplicated in Zotero, and title/abstract and full-text screening will be performed independently by two reviewers using Rayyan, with discrepancies resolved by consensus or adjudication by a third independent reviewer. Data will be charted using a structured Microsoft Excel form and synthesised descriptively in tables and narrative summaries.

## Introduction

An intensive care unit (ICU) is an organised system providing specialist medical and nursing care, continuous monitoring, and life-sustaining organ support during periods of critical illness or organ insufficiency [1]. The PICU provides the same level of care for children with life-threatening diseases requiring organ support.

Survival rates in paediatric intensive care have improved significantly over the past few decades [2]. However, there is growing recognition of the long-term consequences of critical illness, collectively referred to as post-intensive care syndrome (PICS).

Originally defined in adult populations, PICS describes ‘new onset or worsening of impairments in physical, cognitive, and/or mental health that arise after the ICU and persist beyond hospital discharge’ [3]. Children who survive critical illness may also experience persistent deficits across physical, cognitive, emotional, and social domains – now recognised as PICS in paediatrics (PICS-p). These may include difficulties with mobility, feeding, memory, learning, behaviour regulation, or psychological well-being (e.g., post-traumatic stress symptoms), often extending months or years beyond ICU discharge [4].

The impact also extends to families and caregivers, whose lives may be substantially affected by the child’s ongoing needs [5].

Despite increasing awareness, the field of PICS-p remains underdeveloped. Much of the existing literature derives from adult cohorts, limiting direct applicability to children. Paediatric-specific challenges – such as developmental variability, communication barriers, and reliance on caregivers – further complicate assessment and intervention. Consequently, paediatric evidence is fragmented and lacks a unified synthesis.

Given these limitations, a scoping review is warranted to comprehensively map current evidence related to the diagnosis, prevention, and treatment of PICS-p. To capture the full breadth of research, this review focuses on primary studies of post-ICU outcomes among patients aged 1 month–18 years, excluding neonatal-only populations.

This protocol outlines a scoping review that will follow the Joanna Briggs Institute (JBI) methodology and PRISMA-ScR reporting standards. The aim is to synthesise available knowledge on how PICS-p is conceptualised and addressed across different paediatric subpopulations and clinical contexts, and to identify key gaps to inform future research and practice. Findings from this review are expected to inform paediatric intensivists, rehabilitation specialists, psychologists, and policymakers by identifying actionable strategies and knowledge gaps in post-ICU care pathways for children. This may support the development of integrated, family-centred models of follow-up care.

## Research questions

This scoping review will address the following research questions:

1. What diagnostic methodologies are currently used to identify PICS-p in paediatric patients across clinical contexts?
2. What preventive and therapeutic interventions have been implemented to address physical, cognitive, emotional, and social sequelae in children post-ICU?
3. How do these interventions vary across populations, settings, and outcome measures?

## Inclusion criteria

### Participants

This review will include studies involving paediatric patients aged 1 month to 18 years who have survived admission to a paediatric or specialised intensive-care unit. Children with pre-existing congenital or chronic conditions will be included, as these groups constitute a major proportion of paediatric ICU survivors and may experience additional or worsened post-ICU sequelae.

For studies including both paediatric and adult populations, data will be included only if paediatric outcomes are reported separately or can be disaggregated.

Studies limited to neonatal populations (≤ 28 days at admission) or reporting outcomes confined to the neonatal intensive-care context will be excluded. Studies involving caregivers will be included only when the focus is on their role in, or the psychosocial impact of, PICS-p care.

### Concept

The concept of interest is post-intensive-care syndrome in paediatrics (PICS-p**)** – defined as any new or worsening impairment in physical, cognitive, emotional, or social functioning observed after ICU discharge and attributable to paediatric critical illness. Included studies may examine diagnostic or screening methods, preventive or therapeutic interventions, or longitudinal outcomes following paediatric ICU care.

### Context

This review will consider studies from all clinical contexts where paediatric patients are assessed or managed after ICU discharge, including inpatient follow-up programs, outpatient clinics, rehabilitation centres, and community or home settings. No geographic or setting-based restrictions will apply.

### Types of sources

Eligible study types include primary clinical research: prospective or retrospective observational studies, interventional trials, case series, and case reports. Grey literature will also be included if it reports primary clinical data (e.g., preprints, dissertations, registered protocols). Studies will be included regardless of sample size or completeness of outcome reporting; such limitations will be documented in the synthesis. Only studies published in English from January 1, 2000, onwards, will be considered. The 2000–2025 window was selected because the term ‘post-intensive care syndrome’ and related long-term sequelae began gaining clinical and research attention in the early 2000s, particularly following its formal definition in adult populations.

## Exclusion criteria

- Animal or non-human studies
- Studies reporting only theoretical frameworks, expert opinion, or narrative overviews without original clinical data
- Abstract-only records (e.g., conference proceedings without full text)
- Studies focused solely on adult PICS or mixed-age cohorts with no separate paediatric data
- Articles focusing exclusively on PICU outcomes during admission (not post-discharge)
- Studies on routine post-op, trauma, or illness outcomes not explicitly contextualised as PICS-p

## Methods

This scoping review will adhere to the PRISMA-ScR guidelines [6] and the PRISMA-P 2015 checklist [7], further guided by the Joanna Briggs Institute Manual for Evidence Synthesis [8]. Completed PRISMA-ScR (S1 Checklist) and PRISMA-P (S2 Checklist) checklists are available in the supporting information.

The protocol is registered on the Open Science Framework (OSF), in line with best practices for scoping reviews (osf.io/hx3dz). We will employ an adaptive approach, allowing methodological modifications in response to procedural challenges, with all changes transparently documented through updates on the OSF.

Ethics approval is not required, as this study involves secondary analysis of published literature only.

### Search strategy

For peer-reviewed and grey literature, we will systematically search PubMed, Scopus, Cochrane and ProQuest for studies published from 1 Jan 2000 to the date of the search. To further identify grey literature, we will search medRxiv, ClinicalTrials.gov, and the WHO International Clinical Trials Registry Platform (ICTRP). Backward and forward citation chaining of all included studies will be performed to maximise relevant retrieval. All search results will be limited to English-language publications.

To ensure comprehensive coverage across medical, nursing, and allied-health literature, we will additionally search CINAHL, which indexes rehabilitation and family-centred care studies not consistently captured in biomedical databases.

An initial pilot search was conducted in PubMed to identify index terms and keyword variations. Due to the lack of standardised terms for PICS-p, we primarily used free-text terms in titles and abstracts. Search terms were tailored per database and the full strategies are detailed in Table 1.

**Table 1:** Search strategy.

Database	Search Strategy	Limits
PubMed	((“post-intensive care syndrome” OR “PICS-p” OR “post-ICU”) OR ((ICU OR PICU OR CICU OR HICU) AND (outcome OR outcomes OR recovery OR function* OR cognition OR cognitive OR neurodevelopment* OR neuropsych* OR psychological OR mental OR depression OR anxiety OR PTSD OR “quality of life” OR HRQoL OR family OR social OR participation OR wellbeing))) AND (child OR children OR pediatric OR paediatric OR adolescent OR adolescence OR teen OR teenage) NOT (neonate OR neonates OR neonatal OR NICU)	1 Jan 2000 – date of search; English language
Scopus, Cochrane, ProQuest, CINAHL, medRxiv ClinicalTrials.gov, WHO ICTRP	Strategy adapted with identical concept blocks and equivalent Boolean logic.	Same date and language limits

Database-specific syntax (e.g., field tags, truncation) will be adjusted accordingly. Filters for “humans” and “child: 1 month–18 years” will be applied where available.

Two reviewers will independently pilot the search strategies to assess comprehensiveness and reproducibility prior to full execution.

Due to institutional access differences across ProQuest’s databases, search results may vary depending on database availability. To minimise this, all ProQuest searches were conducted from University of Exeter with the same database selection and search filters.

### Sources of evidence and screening

All records from the search will be imported into Zotero for de-duplication, both automatically and manually. Two reviewers will independently screen a pilot sample of 20 title/abstracts, and we will assess inter-reviewer agreement. Disagreements will be discussed and only necessary modifications made to the inclusion and exclusion criteria, to improve inter-reviewer agreement. Any changes will be documented to the OSF. Once inter-rater agreement exceeds 80%, full screening will proceed.

Records will be imported into Rayyan, with independent screening of all full text articles. Reasons for full-text exclusions will be documented. Discrepancies at any stage will first be resolved by discussion between the two reviewers, with adjudication by a third independent reviewer if consensus is not reached. Inter-rater reliability (Cohen’s kappa) will be calculated.

Final selections and exclusions will be reported using a PRISMA flow diagram, which will be used to illustrate the selection process.

### Data extraction

Data will be extracted using a structured Microsoft Excel form. The details extracted will include those outlined in our data extraction chart (Table 2). Two reviewers will independently extract from a random sample of 10 studies to standardise procedures. Discrepancies will be discussed and the data extraction form revised if needed. Any changes to the data extraction chart will be documented on the OSF and in the final review.

**Table 2:** Data extraction variables.

Variable	Description
Citation Details	Author(s), year, journal — for reference tracking.
Study Characteristics	Design (RCT, cohort, case series, etc.); country; publication type (peer-reviewed, grey literature).
Setting/Context	Clinical context (PICU follow-up clinic, rehabilitation, outpatient, community, home care, etc.).
Population Characteristics	Age range, sample size, diagnosis, illness severity, inclusion/exclusion of chronic or congenital conditions.
Follow-up Timing	Months post-discharge and timepoints assessed
PICS-p Domain(s)	Physical, cognitive, psychological, and/or social domains assessed
Diagnostic/ Screening Methods	Tools, scales, or assessments used to identify or monitor PICS-p (e.g., questionnaires, biomarkers, imaging, functional tests).
Intervention Characteristics	Type (rehabilitation, psychological therapy, education, family support, etc.); target domain; duration; mode of delivery.
Outcomes Measured	Key findings related to recovery, symptom burden, HRQoL, functional improvement, school re-entry, or family outcomes.
Effectiveness/ Impact Summary	Summary of reported effects or improvements; any measured associations or trends.
Gaps and Limitations	Limitations in design or data – missing data, methodological weaknesses, underrepresented domains or populations.
Reviewer Notes	Observations, decisions during extraction, or points to follow up during synthesis.

Each reviewer’s data extraction form will be compared, with disagreements discussed and resolved through consensus. A third independent reviewer will adjudicate any unresolved differences.

No formal quality appraisal will be conducted in keeping with scoping review methodology, but limitations in reporting and design will be noted.

A finalised chart will be produced, for use in our data analysis and synthesis of results.

### Synthesis of results

Findings will be summarised in tables and narratively, related to our research questions. Where appropriate, results will be synthesised thematically by settings and timing of follow-up, clinical assessments performed (e.g., screening tools, biomarkers, imaging), interventions trialed (e.g., psychology, rehab, family support), and targeted outcomes (e.g., quality of life, neurodevelopment, PTSD, school re-entry).

No meta-analysis will be performed due to anticipated heterogeneity in design, population, and outcome measures.

### Study timeline

The scoping review is expected to take approximately 12–16 weeks. At the time of submission, no stages of the review have been completed, including record screening, data extraction, or data synthesis. No participant recruitment or primary data collection will take place, as these are not applicable to this study, where we will be extracting data based on existing research and literature. The planned timeline for the review is as follows: 2–3 weeks for record screening, 3–4 weeks for data extraction, 3 weeks for reviewing and summarising findings, and 4–5 weeks for manuscript drafting. The study will begin after acceptance for peer review, pending any revisions that may arise from the review process. We anticipate that the results will be ready within 4 months after study initiation.

## Discussion of strengths, limitations and impact

This scoping review has several key strengths. It represents the first structured synthesis focused specifically on the clinical management strategies for paediatric post-intensive care syndrome (PICS-p), addressing a critical gap in the literature. The protocol adheres to established methodological frameworks, including PRISMA-ScR and the Joanna Briggs Institute (JBI) guidelines, ensuring a rigorous and transparent approach. Additionally, the inclusion of grey literature and trial registries enhances the comprehensiveness of the evidence base and helps mitigate publication bias.

The incorporation of citation tracking will maximise retrieval of relevant literature, ensuring comprehensive coverage.

However, there are also some limitations to consider. The restriction to English-language sources introduces potential language bias, and the reliance on keyword-based search strategies may result in the omission of relevant studies that only reference PICS-p in the full text. Furthermore, in keeping with the nature of scoping reviews, no formal risk of bias assessment will be conducted, which may limit the ability to evaluate the quality of the included evidence.

By restricting the population to children and adolescents aged > 1 month–18 years and excluding neonatal-only cohorts, this review provides a focused synthesis of paediatric post-ICU survivorship while acknowledging that separate neonatal follow-up literature exists.

A comprehensive scoping of this topic will encourage paediatric intensivists, rehabilitation specialists, psychologists, and policymakers to strategically consider the full package of care for critically ill children. A study of this nature may be a prerequisite for integrated, family-centred models of follow-up care.

## Supporting information

S1 Checklist PRISMA-ScR checklist. The checklist used to ensure reporting standards for the scoping review.
(PDF)

S2 Checklist PRISMA-P checklist. The checklist used to ensure reporting standards for the protocol.
(PDF)
